# Performance of large language models on veterinary undergraduate multiple-choice examinations: a comparative evaluation

**DOI:** 10.3389/fvets.2025.1616566

**Published:** 2025-08-26

**Authors:** Santiago Alonso Sousa, Syed Saad Ul Hassan Bukhari, Paulo Vinicius Steagall, Paweł M. Bęczkowski, Antonio Giuliano, Kate J. Flay

**Affiliations:** ^1^Department of Veterinary Clinical Sciences, Jockey Club College of Veterinary Medicine and Life Sciences, City University of Hong Kong, Kowloon, Hong Kong SAR, China; ^2^Centre for Animal Health and Welfare, City University of Hong Kong, Kowloon, Hong Kong SAR, China

**Keywords:** veterinary education, artificial intelligence, assessment, large language models, comparative analysis, quality assurance

## Abstract

The integration of artificial intelligence, particularly large language models (LLMs), into veterinary education and practice presents promising opportunities, yet their performance in veterinary-specific contexts remains understudied. This research comparatively evaluated the performance of nine advanced LLMs (ChatGPT o1Pro, ChatGPT 4o, ChatGPT 4.5, Grok 3, Gemini 2, Copilot, DeepSeek R1, Qwen 2.5 Max, and Kimi 1.5) on 250 multiple-choice questions (MCQs) sourced from a veterinary undergraduate final qualifying examination. Questions spanned various species, clinical topics and reasoning stages, and included both text-based and image-based formats. ChatGPT o1Pro and ChatGPT 4.5 achieved the highest overall performance, with correct response rates of 90.4 and 90.8% respectively, demonstrating strong agreement with the gold standard across most categories, while Kimi 1.5 showed the lowest performance at 64.8%. Performance consistently declined with increased question difficulty and was generally lower for image-based than text-based questions. OpenAI models excelled in visual interpretation compared to previous studies. Disparities in performance were observed across specific clinical reasoning stages and veterinary subdomains, highlighting areas for targeted improvement. This study underscores the promising role of LLMs as supportive tools for quality assurance in veterinary assessment design and indicates key factors influencing their performance, including question difficulty, format, and domain-specific training data.

## Introduction

The role of artificial intelligence (AI) in healthcare has become a prominent focus in recent scholarly discussions (1–4). This attention is driven by rapid advancements in large language models (LLMs), a subset of AI systems capable of generating human-like natural language responses from textual input (3). Among current LLMs, ChatGPT, a chat-generative pre-trained transformer developed by OpenAI, has emerged as especially relevant due to its sophisticated deep-learning architecture, trained on extensive datasets, enabling it to produce coherent, contextually appropriate responses to user prompts (5, 6). ChatGPT demonstrates capabilities beyond knowledge recall, with reports of deductive reasoning and chain of thought (CoT) (5). This facilitates broader applications of ChatGPT (and other comparable AI tools) within the medical field, such as answering medical questions (7), writing medical reports (8), information retrieval (9), aiding study (6, 10) and professional development (11). In 2022, with no specialized training, ChatGPT performed at or near the United States Medical Licensing Exam (USMLE) passing threshold of 60% accuracy (12). Apart from that, ChatGPT has passed general medical licensing examinations from Australia (13), Peru (14) and Iran (15), and has also exceeded passing scores in sub-specialties such as dermatology (16) and radiology (17).

Going forwards, LLMs are predicted to impact all aspects of society, including education and the training and assessment of healthcare professionals (18–20). Recently, more LLMs such as Grok, DeepSeek or Copilot, have shown strong performance in effectively addressing a wide range of queries (21). However, all these LLMs have shown varying proficiency across different healthcare disciplines (21–26), and additional concerns have been raised regarding their understanding of questions, depth of responses and ability to deal with nuanced and context dependent data (2, 11, 27).

Currently, our understanding of the capabilities of LLMs in veterinary science is limited. Few studies have specifically examined the performance of ChatGPT within this context (28–30), and therefore conclusive evidence regarding the accuracy of LLMs in answering veterinary-specific examination questions is lacking. Accuracy in clinical decision-making, whether in human or veterinary medicine, is paramount, as even minor inaccuracies may lead to serious clinical consequences. Given that LLMs primarily function as language-generation systems rather than structured knowledge bases, concerns regarding the accuracy and reliability of their outputs are particularly critical (31, 32). Furthermore, comparative evaluations of LLMs have predominantly focused on human medicine or related healthcare disciplines, with veterinary medicine largely excluded from these assessments (26).

In veterinary education, veterinary graduates are required to demonstrate proficiency across multiple domains, including knowledge, problem-solving, clinical skills, communication, and professionalism (33–35). Although various assessment methods are simultaneously employed to evaluate competence holistically, multiple-choice questions (MCQs) remain extensively utilized as a summative assessment format (36–38). Notably, MCQs constitute the primary format of the North American Veterinary Licensing Examination (NAVLE; International Council for Veterinary Assessment, 2025) (39). Such examinations typically emphasize clinical decision-making, a multifaceted cognitive process that integrates veterinary knowledge, clinical reasoning, the ability to synthesize information from diverse species, and the capacity to apply evidence-based practices.

In this study, we aimed to investigate the potential of LLMs in answering MCQs in veterinary knowledge according to species, subject, type of MCQ (image vs. text-based), clinical reasoning and difficulty levels. The hypothesis was that LLMs would present different accuracy and that would change according to the type of question (species, subject, type of MCQ, clinical reasoning and difficulty levels).

## Materials and methods

### Large language models

Nine widely recognized LLMs were evaluated in this study between January and February 2025. The models assessed were: (1) ChatGPT o1Pro (OpenAI), (2) ChatGPT 4o (OpenAI), (3) ChatGPT 4.5 (OpenAI), (4) Grok 3 (xAI), (5) Gemini 2 (Google), (6) Copilot (Microsoft), (7) DeepSeek R1 (DeepSeek), (8) Qwen 2.5 Max (Alibaba Cloud), and (9) Kimi 1.5 (Moonshot AI). These specific versions were chosen because they represented the most advanced and updated iterations available during the study period, optimized for reasoning capabilities. Models from OpenAI and xAI were accessed via paid subscription services, whereas the other models were freely accessible to the public at the time of the evaluation.

### Multiple-choice questions design and inclusion

The MCQs utilized in this study were derived from the final qualifying examination for the Bachelor of Veterinary Medicine (BVM) Program at the City University of Hong Kong. A total of 250 MCQs were included, each structured as a clinical vignette with a single best answer selected from five options, comprising one correct response and four plausible distractors. These questions were developed to assess knowledge and cognitive skills expected from Day 1 veterinary graduates, aligning with international competency frameworks such as the Australasian Veterinary Boards Council (AVBC) Attributes (35), Royal College of Veterinary Surgeons (RCVS) Day One Competences (33), and the World Organization for Animal Health (WOAH) Competencies of Graduating Veterinarians (40).

The MCQs underwent external benchmarking with peer institutions from the United Kingdom, Australia, and the United States, mirroring the format of the NAVLE. The inclusion and selection criteria for MCQs included reliability, reproducibility, fairness, objectivity, credibility, simplicity of administration, and potential for facilitating constructive feedback.

Content validity was ensured through a comprehensive competency-based educational blueprint, created by three independent expert groups consisting of five subject matter experts each, aligning closely with NAVLE guidelines. This blueprint accounted for the relative importance and instructional hours dedicated to each subject area and animal species within the veterinary curriculum.

MCQ writers, including veterinary specialists and faculty members, received training in best practices for MCQ development. Initial MCQ drafts underwent critical review and individualized feedback from external experts in veterinary education, ensuring adherence to established quality standards. Questions identified as non-compliant were revised and resubmitted until they met the quality criteria. Reliability was investigated through beta-testing by qualified veterinarians (faculty and teaching staff), accompanied by psychometric analysis, including item facility, discrimination indices, and point-biserial correlation, conducted using Speedwell examination software (Speedwell Software Ltd., United Kingdom).

The standard-setting process was conducted using the modified Angoff method (41), where a panel of veterinarians (i.e., instructors) with diverse subject matter expertise, including specialists and general practitioners, were trained on standard-setting principles and inter-rater agreement before establishing the minimum passing score. This criterion-based pass mark ensured fairness and objectivity, independent of candidate group performance. Post-examination psychometric analyses verified the ongoing validity, reliability, and alignment with the veterinary competency framework.

For analytical purposes, MCQs were further classified according to their difficulty levels (low difficulty [≥70% of instructors expect students to answer correctly]), (medium difficulty [40–69% of instructors expect students to answer correctly]), (high difficulty ≤39% of instructors expect students to answer correctly); species (Canine, Feline, Equine, Bovine, Other Production Animals [including small ruminants and swine], and Exotics [including birds, reptiles, amphibians, and small mammals]); image or text-based questions; clinical reasoning stages (e.g., diagnostic interpretation, diagnostic plan, treatment/prognosis, clinical assessment, and prophylaxis); and specific veterinary subdomains (e.g., cardiology, oncology, dentistry, anesthesia/pain management, diagnostic imaging, behavior, and soft tissue surgery) for subsequent analysis.

### LLM performance

To ensure that the evaluation was unaffected by prior interactions, each LLM chatbot was assessed using a newly created account with no previous conversation history. A standardized, structured prompt (*“Which of the following is the most appropriate answer for this question: 1, 2, 3, 4 or 5?”*) was consistently inputted into each AI model, followed by clear separation before presenting the MCQs and their corresponding options numbered from 1 to 5. The structured prompt was specifically designed to maintain consistency across chatbot interactions and minimize bias. The MCQs required selecting only the best answer from the given options, without providing any additional justification. Several questions included visual elements (images), which were directly attached as JPG files within the chatbot interface. MCQs were systematically delivered in batches of five to prevent potential chatbot overload or context loss. Each chatbot’s responses were documented via manual transcription for subsequent analysis.

### Statistical analysis

Responses provided by each chatbot were compared to the gold standard (correct answer sheet), and performance was expressed as agreement percentages (%). Cohen’s kappa coefficient (*κ*) (42, 43) was calculated to assess overall agreement between each chatbot and the gold standard. Further analyses using Cohen’s kappa coefficient were conducted to evaluate agreement within specific subcategories, including question difficulty levels (low, medium, and high), species, type of clinical reasoning required, presence or absence of images, and clinical categories. Cohen’s kappa values were interpreted following established guidelines: 0–0.20 indicating no agreement, 0.21–0.39 minimal, 0.40–0.59 weak, 0.60–0.79 moderate, 0.80–0.90 strong, and >0.90 almost perfect agreement (42, 43). All statistical analyses were performed using RStudio version 2022.07.1–554 (44).

## Results

In general, ChatGPT o1 Pro and ChatGPT 4.5 models had the highest agreement rate, followed by Copilot, DeepSeek R1 and ChatGPT 4o, all showing a strong agreement level. None of the models achieved almost perfect agreement (Cohen’s kappa values of >0.90). Kimi 1.5 model performed the worst with a weak level of agreement, and a rate of correct responses of only 64.8% (Table 1).

**Table 1:** Agreement percentages (performance) of different LLMs compared to the gold standard (correct answers) on 250 veterinary MCQs.

Chatbot	Correct answers (%)	Cohen’s kappa coefficient
ChatGPT o1 Pro	90.4	0.88
ChatGPT 4o	85.5	0.81
ChatGPT 4.5	90.8	0.88
Grok 3	79.2	0.73
Gemini 2	77.2	0.71
Copilot	85.6	0.82
DeepSeek R1	85.6	0.82
Qwen2.5 Max	79.6	0.74
Kimi 1.5	64.8	0.56

Results are presented as percentages (%) of correct answers and Cohen’s kappa coefficients (*κ*) for overall agreement; 0–0.20 indicating no agreement, 0.21–0.39 minimal, 0.40–0.59 weak, 0.60–0.79 moderate, 0.80–0.90 strong, and >0.90 almost perfect agreement.

### Agreement rate between LLMs according to level of difficulty

**Figure 1:**
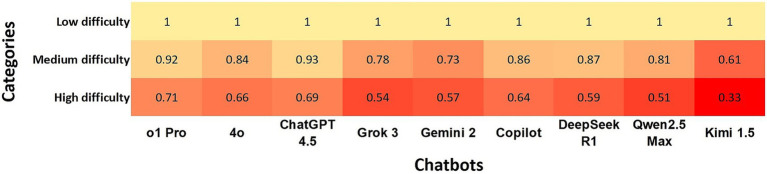
Heatmap shows the agreement of chatbots with the gold standard using Cohen’s kappa coefficients at questions defined as low, medium, and high difficulty levels. In the heatmap, a darker box color indicates lower agreement, while a lighter color indicates higher agreement.

There was almost perfect agreement between all chatbots and the gold standard at low difficulty level (*κ* = 1.0). At a medium difficulty level, the agreement from ChatGPT o1Pro and ChatGPT 4.5 remained almost perfect; however, agreement was strong to the other chatbots, except Grok 3, Gemini 2 and Kimi 1.5, which presented moderate agreement. At a high-level difficulty ChatGPT o1PRO, ChatGPT 4.5, ChatGPT 4o and Copilot showed moderate agreement whereas Kimi 1.5 presented minimal agreement (Figure 1).

### Agreement rate between LLMs based on image-based and non-image-based questions

The agreement between OpenAI chatbots and the gold standard was strong for both text-based and image-based questions. In contrast, Kimi 1.5 showed weak agreement with the gold standard, achieving *κ* = 0.46 for image-based questions and κ = 0.57 for text-based questions (Table 2). Although the categorical agreement levels for each LLM did not differ between image-based and text-based questions, overall performance tended to be lower on questions containing images.

**Table 2:** Agreement of chatbots with the gold standard for photo and non-photo questions.

Chatbots	Cohen’s kappa coefficients for image-based questions	Cohen’s kappa coefficients for non-image-based questions
ChatGPT o1 Pro	0.82	0.88
ChatGPT 4o	0.82	0.81
ChatGPT 4.5	0.81	0.89
Grok 3	0.63	0.75
Gemini 2	0.62	0.74
Copilot	0.72	0.79
DeepSeek R1	0.78	0.79
Qwen2.5 Max	0.72	0.74
Kimi 1.5	0.46	0.57

The values in the table represent Cohen’s kappa coefficients (κ).

### Agreement rate of chatbots with the gold standard according to species

**Figure 2:**
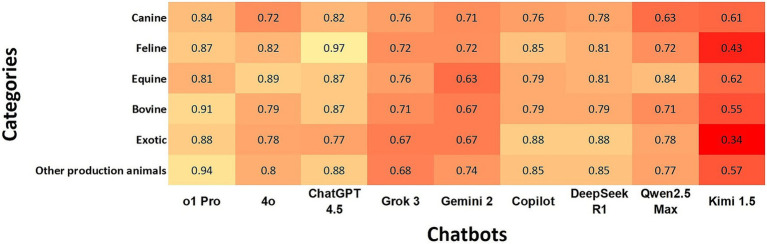
Heatmap shows the agreement of chatbots with the gold standard using Cohen’s kappa coefficients according to species. In the heatmap, a darker box color indicates lower agreement, while a lighter color indicates higher agreement.

ChatGPT o1Pro showed almost perfect agreement with “Bovine” and “Other Production Animals,” while ChatGPT 4.5 exhibited almost perfect agreement with “Feline.” Both chatbots maintained strong agreement levels with the other species. DeepSeek R1 presented strong agreement with four species. In contrast, Kimi 1.5 had the lowest performance, showing minimal agreement with “Exotic” and weak agreement with three species (Figure 2). When comparing species, “Feline” and “Other Production Animals” demonstrated the strongest agreement rates among the chatbots, while “Canine” and “Exotics” categories had the lowest agreement rates (Figure 2).

### Agreement rate of chatbots with the gold standard according to clinical reasoning

**Figure 3:**
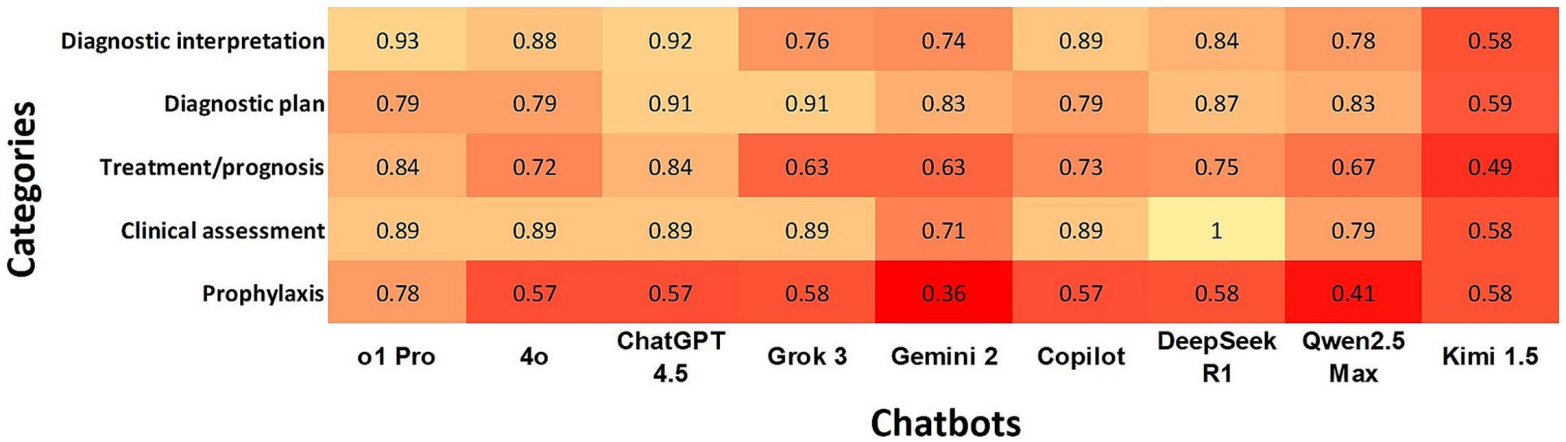
Heatmap shows the agreement of chatbots with the gold standard using Cohen’s kappa coefficients according to clinical reasoning. In the heatmap, a darker box color indicates lower agreement, while a lighter color indicates higher agreement.

ChatGPT o1Pro, ChatGPT 4.5, and DeepSeek R1 demonstrated the highest overall agreement rates with the gold standard across all categories, while Kimi 1.5 exhibited the lowest overall agreement. “Diagnostic Interpretation” and “Diagnostic Plan” showed the highest agreement rates among chatbots, whereas “Prophylaxis” had the lowest level of agreement (Figure 3).

### Agreement rate of the chatbots with the gold standard according to subdomains

ChatGPT o1Pro and ChatGPT 4.5 had almost perfect agreement in nine and eight subdomains, respectively. Copilot and DeepSeek R1 showed perfect agreement in six subdomains. Conversely, Kimi 1.5 had minimal agreement in six subdomains and non-agreement in one.

When evaluating agreement by subdomains, the highest overall agreement percentages were observed in Endocrinology/Metabolic Diseases, Cardiology, Oncology, and Ophthalmology. In contrast, the lowest agreement levels occurred in Dentistry, Behavior, Anesthesia/Pain Management, Diagnostic Imaging, and Soft Tissue Surgery.

## Discussion

In this study, we evaluated the performance of nine LLMs using a dataset of MCQs sourced from the final qualifying examination for the BVM Program at the City University of Hong Kong. To our knowledge, this represents the first comparative evaluation of LLMs targeting veterinary clinical knowledge. Notably, ChatGPT o1Pro and ChatGPT 4.5 outperformed the other chatbots assessed in our study. These results may be explained by OpenAI’s recent efforts to enhance deductive reasoning and critical judgment capabilities through advanced prompting techniques such as CoT (45, 46). CoT prompts allow these models to systematically deconstruct complex problems, enhancing their ability to interpret questions accurately and provide precise answers.

DeepSeek R1 achieved agreement rates similar to ChatGPT 4o and Copilot, the latter being based on ChatGPT 4o’s architecture, especially considering its relatively recent development. This comparable performance is particularly notable given differences in the underlying architecture and training approaches of these models. OpenAI utilizes reinforcement learning from human feedback (RLHF), a technique where models are fine-tuned using human-generated reward signals to optimize output quality and alignment. This approach is combined with supervised fine-tuning (SFT), where models learn from labeled datasets containing input–output pairs provided by human annotators. These methods are employed in their advanced models during the pre-training phase. In contrast, DeepSeek integrates reinforcement learning and SFT applied to pre-trained data within a mixture-of-experts architecture (47). On the other hand, Kimi 1.5 had weak agreement and the lowest performance when compared with the other LLMs. In previous studies, Kimi 1.5 performed better than ChatGPT 4o and equal to ChatGPT o1 (48) with respect to reasoning; however, our study did not corroborate these findings. This may be due to the build-in model architecture. Kimi’s reinforcement learning (RL) approach avoided questions such as MCQs, because these could be answered correctly without good reasoning which could hack the reward model, potentially leading to a possible worse performance with MCQs (48).

High accuracy of LLMs in veterinary knowledge has become increasingly important as a growing number of pet owners rely on these platforms for guidance regarding their pets’ health (49). Although these systems can enhance pet owners’ general knowledge and understanding, they also carry significant risks of misinformation, which may lead to detrimental outcomes (50). Our study, which evaluated some of the newest LLMs known for advanced reasoning capabilities, yielded findings consistent with previous research that compared ChatGPT 3.5 and ChatGPT 4 in answering MCQs and true/false questions, reporting accuracy rates of 55 and 77%, respectively (30).

When comparing the agreement levels of different LLMs with the gold standard across low, medium, and high difficulty MCQs, our results indicate that all models achieved almost perfect agreement at low difficulty levels. However, agreement levels decreased as question difficulty increased. ChatGPT o1Pro and ChatGPT 4.5 maintained a moderate level of agreement even at higher difficulty levels. These findings align with previous research showing that ChatGPT’s performance on the United States Medical Licensing Examination declined as question difficulty increased (7). Our results also parallel higher LLM performance on the primary-care-level National Certificate Examination for Primary Diabetes Care in China compared to specialist-level Specialty Certificate Examination in Endocrinology and Diabetes administered by the Royal College of Physicians of the United Kingdom (21). This decline in performance at higher difficulty levels is likely due to limitations in the models’ logical reasoning capabilities. As questions become more challenging, correct answers depend on domain-specific knowledge and sound logical reasoning capabilities, which can become strained when cognitive demands exceed the model’s current training and reasoning capacities (7).

A distinctive aspect of our study is the inclusion of both text-based and image-based questions, reflecting the integral role of visual assessment in veterinary practice. LLMs had similar agreement levels when comparing text and image-based questions. However, there was an overall lower performance with image-based questions using almost all models. OpenAI models were the only LLMs to consistently achieve a strong agreement level across both question types. These findings align with other healthcare studies, which similarly observed decreased performance of LLMs on image-based questions compared to text-based questions (51, 52). However, when comparing our results to previous evaluations of OpenAI models, our study demonstrates a significant improvement. The agreement performance increased from 57% on image-based questions in a study assessing ChatGPT 4 on the Fellowship of the Royal College of Surgeons Trauma and Orthopedics examination (53) to 89% accuracy with OpenAI models in our study. This notable advancement could indicate a substantial progress in these models’ capacity to analyze and interpret image-based data, a critical skill, particularly in specialties such as diagnostic imaging. The observed improvement likely results from enhanced training techniques and larger, more diverse visual datasets, enabling AI models to learn effectively from a broader spectrum of visual inputs (54, 55).

**Figure 4:**
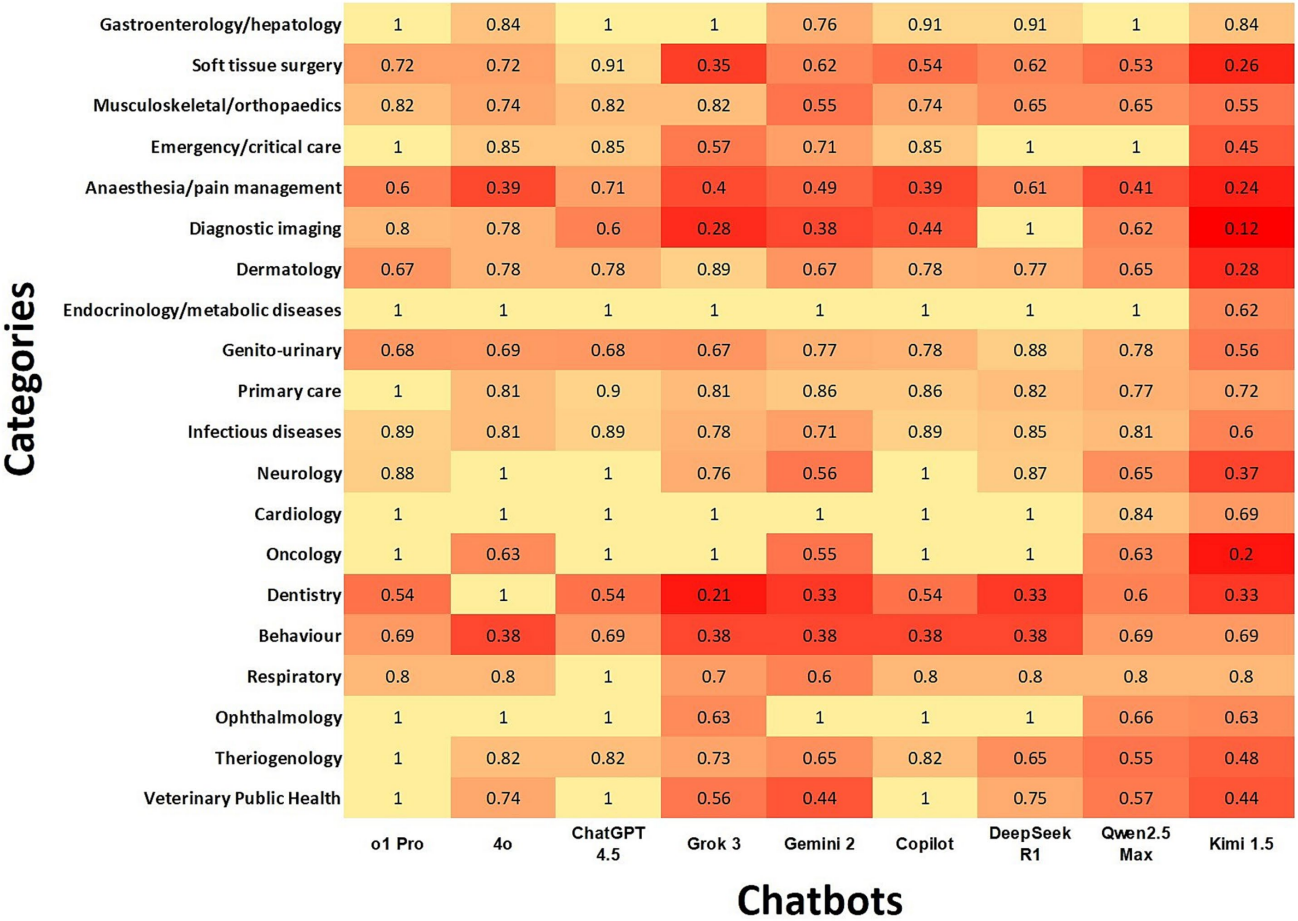
Heatmap illustrates chatbot agreement with the gold standard using Cohen’s kappa coefficients according to subdomains. In the heatmap, darker color indicates lower agreement, while lighter color represents higher agreement.

While our study highlights the strong performance of multiple LLMs across various veterinary domains, we also observed inconsistencies across clinical reasoning (Figure 3) and subdomains (Figure 4). Specifically, within clinical reasoning, the areas of treatment/prognosis and prophylaxis demonstrated the lowest levels of agreement. Additionally, subdomains including dentistry, behavior, anesthesia/pain management, soft tissue surgery, and diagnostic imaging exhibited comparatively lower agreement levels across all LLMs. Several factors may explain these discrepancies. First, the broad and interdisciplinary nature of these subdomains can introduce ambiguity due to extensive and overlapping content across multiple species. Second, rapid advancements in specialized areas such as anesthesia, pain management, and soft tissue surgery may result in knowledge gaps if the training datasets of these LLMs are not sufficiently up-to-date. Third, decreased performance in diagnostic imaging aligns with our earlier findings that LLMs exhibit inherent limitations in accurately analyzing and interpreting image-based data. Additionally, although LLMs are trained on extensive datasets encompassing various domains, human health represents a significantly larger portion of available data compared to veterinary medicine (30). This disproportionate focus on human medical data could contribute to disparities in accuracy between veterinary and human health responses (30). Finally, it is possible that these questions require additional internal audit. Our findings suggest that despite the demonstrated potential of certain LLMs in veterinary medicine, further targeted training and model development are essential to achieve consistent and reliable performance across all veterinary subjects.

Our evaluation of LLM performance relied on MCQs, a format known to often contain inherent imperfections known as item-writing flaws (IWFs) (56). Although flaws may seem minor, they can influence how learners interpret and respond to questions, potentially leading to misleading results (57, 58). IWFs could partially explain why, out of the 250 MCQs used in our study, there were six questions that all evaluated LLMs answered incorrectly, and 21 questions where more than 50% of the models failed to provide correct answers. Upon subsequent individual assessment by faculty authors, we determined that several of these incorrectly answered questions did indeed contain IWFs, potentially creating ambiguous or misleading scenarios. The occurrence of IWFs in MCQs can often stem from differences in academic training, variation in clinical expertise among educators in question-writing practices (59), or from constraints such as insufficient time to adequately develop high-quality MCQs (60). Thus, enhancing MCQ quality through targeted interventions appears valuable. This challenge also creates an opportunity for integrating LLMs into veterinary education as supportive tools for quality assurance and internal audit, helping educators with the identification and revision of MCQs that warrant additional scrutiny (61).

This study has several limitations. First, we evaluated nine LLMs available at the time of the study. Given the rapid evolution and continuous updates of LLM technology, our findings may not fully represent the latest performance of these or other models. Second, our dataset comprised a limited sample size of 250 MCQs, distributed across six veterinary species, potentially insufficient to comprehensively represent the extensive knowledge base of LLMs. Third, we did not have access to the average scores of veterinary students who took the original examination, which prevented direct comparison of student performance with LLMs outcomes. Fourth, although MCQs are widely utilized to assess foundational knowledge, we did not evaluate the reasoning processes underlying the models’ decision-making. Consequently, our study does not reflect the models’ capabilities in handling complex, open-ended clinical scenarios. Lastly, as indicated in prior research, the reproducibility of answers provided by LLMs has been inconsistent, resulting in variable responses upon repeated questioning (62). Nevertheless, recent studies have noted significant improvements in reproducibility among newer LLMs (63). Furthermore, previous studies have demonstrated that LLM performance significantly decreases when responding to prompts in languages other than English, likely due to the dominance of English-language data in their training datasets (64). Consequently, the findings presented here may not directly extrapolate to multilingual veterinary education settings, highlighting the need for future research to assess LLM accuracy across different languages and to enhance multilingual support for equitable global use.

In conclusion, this comparative evaluation of LLMs highlights their varied strengths and weaknesses across different veterinary domains, with ChatGPT o1Pro and ChatGPT 4.5 demonstrating the strongest overall performance. Most of the evaluated LLMs exhibited improved accuracy compared to previous veterinary-focused studies. Key factors influencing performance included question difficulty, with higher complexity significantly reducing model accuracy, and question format, with image-based questions generally yielding lower performance than text-based ones. These findings highlight the potential role of LLMs as valuable supportive tools in veterinary education, particularly for quality assurance in assessment design and implementation.

## Data Availability

The data analyzed in this study is subject to the following licenses/restrictions: confidentiality. Requests to access these datasets should be directed to salonsos@cityu.edu.hk.
